# The association of increased SNAP benefits during COVID-19 with food insufficiency and anxiety among US adults: a quasi-experimental study

**DOI:** 10.1017/S1368980024001447

**Published:** 2024-09-27

**Authors:** Kaitlyn E Jackson, Amy Yunyu Chiang, Rita Hamad

**Affiliations:** 1 Harvard T.H. Chan School of Public Health, Department of Social & Behavioral Sciences, Boston, MA, USA; 2 Philip R. Lee Institute for Health Policy Studies, University of California San Francisco, San Francisco, CA, USA

**Keywords:** Supplemental Nutrition Assistance Program (SNAP), COVID-19, Food insecurity, Mental health, Economic security, Difference-in-differences, Policy evaluation

## Abstract

**Objectives:**

The COVID-19 pandemic and subsequent policy response to mitigate disease spread had far-reaching impacts on health and social well-being. In response, the Supplemental Nutrition Assistance Program (SNAP) underwent several pandemic-era modifications, including a 15 % monthly benefit increase on January 1, 2021. Research documenting the health effects of these SNAP modifications among low-income households and minoritized groups who were most impacted by the economic fallout during the first years of the pandemic is lacking. We aimed to estimate the health effects of the 15 % SNAP benefit increase in January 2021, among SNAP-eligible US households.

**Design:**

We estimated the effects of the SNAP increase on food insufficiency, mental health, and financial well-being using a rigorous quasi-experimental difference-in-differences (DID) analysis.

**Setting:**

August 19, 2020, to March 29, 2021.

**Participants:**

Participants were drawn from the national US Census Bureau Household Pulse Survey waves 13–27 (*n* 44 477).

**Results:**

Compared with SNAP-eligible non-recipients, SNAP-eligible recipients experienced decreased food insufficiency (–1·9 percentage points (pp); 95 % CI –3·7, –0·1) and anxiety symptoms (–0·09; 95 % CI –0·17, –0·01), and less difficulty paying for other household expenses (–3·2 pp; 95 % CI –4·9, –1·5) after the SNAP benefit increase. Results were robust to alternative specifications.

**Conclusions:**

Expansions of federal nutrition programmes have the potential to improve health and financial well-being. This study provides timely evidence to inform comprehensive safety net nutrition policies during future economic crises and public health preparedness response plans.

The COVID-19 pandemic and subsequent policy response to mitigate disease spread have had far-reaching impacts on health and social well-being. This included sharp and sustained increases in unemployment, economic hardship, and food insecurity and declines in mental health^(1–6)^. Effects were disproportionately experienced by families with children, low-income individuals, and communities of colour^(7,8)^. Data from the US Census Bureau Household Pulse Survey (HPS) reported that in June 2020, 8–10 % of US households were experiencing food insufficiency, while prevalence of depressive and/or anxiety symptoms among US adults was three-fold higher than 2019 baseline levels^(5,6)^. Rapid local and federal policymaking aimed to curb the spread of COVID-19 (e.g. shelter-in-place orders), while also addressing the social, economic and health consequences of the pandemic (e.g. the Families First Coronavirus Response Act and Coronavirus Aid, Relief, and Economic Security Act)^(9)^.

The Supplemental Nutrition Assistance Program (SNAP, formerly known as food stamps) provides over 41 million low-income American households with a monthly cash benefit in the form of an Electronic Benefits Transfer (EBT) card to purchase food^(10,11)^. During the pandemic, SNAP underwent several modifications, including increases in benefits from Emergency Allotments (EA) (Fig. 1)^(12–14)^. One of the largest EA expansions, which affected all SNAP recipients, involved a 15 % increase in monthly cash benefits above the maximum benefit for all recipients implemented January 1, 2021^(9,11,15)^. This EA differed from the first EA in March 2020 in that it led to an *additional* 15 % increase for *all* SNAP recipients. As Fig. 1 shows, there were also standard annual modifications to SNAP that took place during the pandemic period in the form of Thrifty Food Plan cost-of-living adjustments in October 2021. SNAP maximum benefits are determined in June of each year by the Thrifty Food Plan and take effect October 1. These Thrifty Food Plan modifications are subject to change each year and often differentially affect SNAP-eligible households based on household size. Prior to the pandemic, a single parent with two children not yet eligible for the household maximum received $449 per month in SNAP benefits. After the 15 % increase above the maximum benefit in January 2021, this amount increased to $616 per month until the SNAP EAs expired^(12,16)^. Recent evidence from one study found that the 15 % SNAP increase prevented 850 000 weekly instances of food insufficiency and decreased food pantry visits among SNAP recipients from January through August 2021^(17)^. Prior to the COVID-19 pandemic, studies found that SNAP helped families afford adequate food, reduced food insecurity, and was a highly effective form of economic stimulus during previous recessions^(18,19)^.

**Fig. 1:**
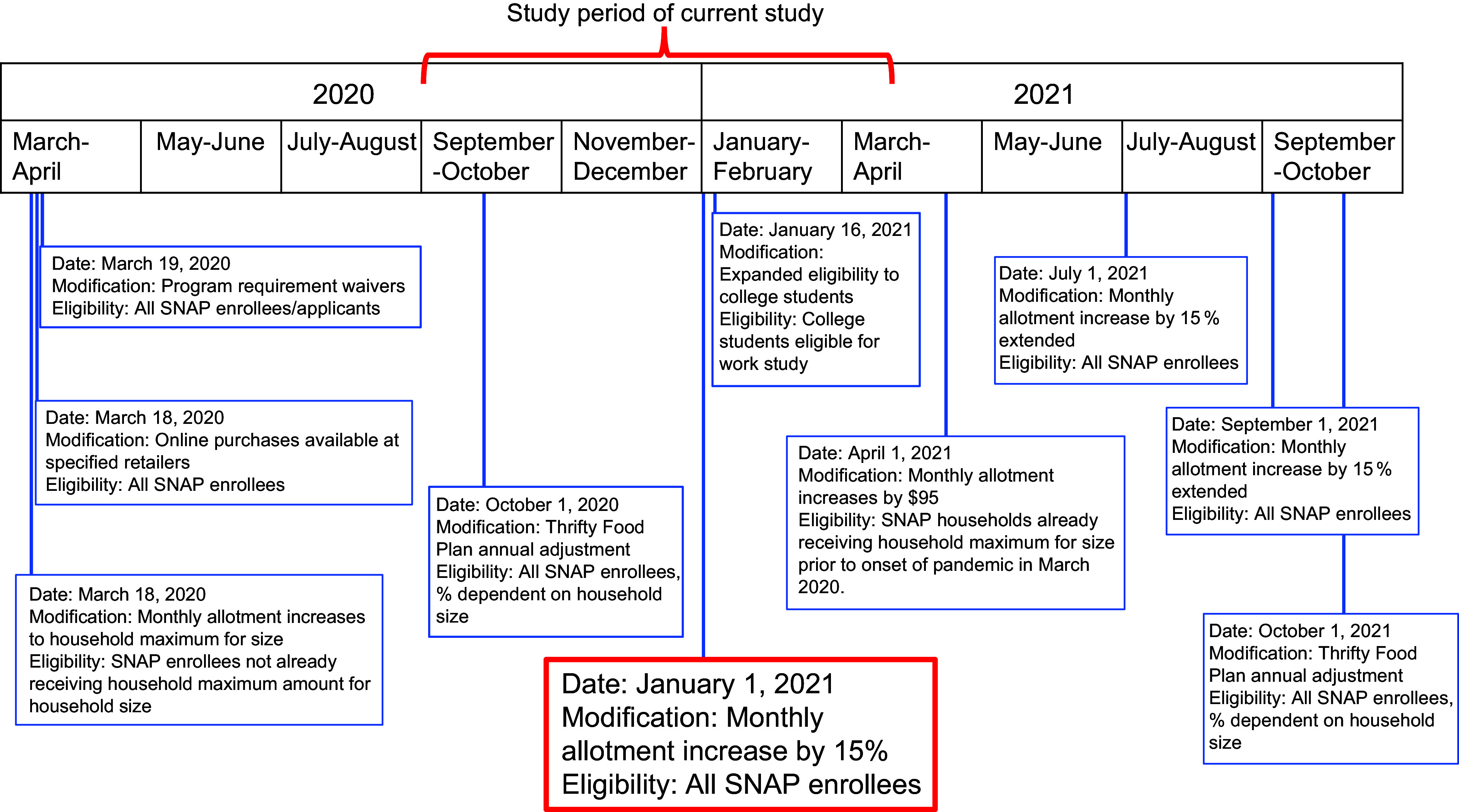
Timeline of SNAP programme modifications, March 2020 through October 2021. SNAP, Supplemental Nutrition Assistance Program

Food insufficiency and financial insecurity are shown to be key determinants of mental health problems (Fig. 2), and pandemic-era research suggests that this relationship was further amplified during the crisis^(2,5)^. Despite the well-studied relationship between food insufficiency, financial well-being, and mental health, studies on the specific effects of pandemic-era SNAP benefit increases on additional health and economic outcomes are limited^(17,20)^. Moreover, prior work suggests that SNAP benefits allow families to spend more on other non-food items, but this has not been examined in the context of this recent benefit increase^(21)^. Previous findings also show that safety net programmes may have differential effects among various subgroups because of differences in underlying risk factors, take-up, or other contributing factors^(22)^.

**Fig. 2:**
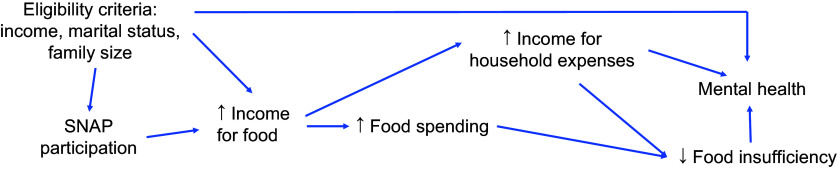
Potential pathway linking SNAP enrolment to financial well-being, food insufficiency and mental health. SNAP, Supplemental Nutrition Assistance Program.

The present study addressed these knowledge gaps by estimating the effects of the 15 % pandemic-era SNAP benefit increase on food insufficiency, mental health, and markers of financial distress among SNAP-eligible Americans using a rigorous quasi-experimental approach. Given previously documented racial, ethnic, and sociodemographic disparities in food insecurity, mental health, and financial hardship brought on by the pandemic, this study also estimated whether effects differed among key subgroups^(7,8)^. Although a permanent SNAP benefit increase was implemented in October 2021 after programme cost-of-living adjustments, the economic consequences of the pandemic have persisted and inflation continues to increase food prices^(12)^. Furthermore, in March 2023, all fifty states had ended SNAP pandemic-era programme expansions, some expiring as early as March 2021^(23,24)^. Evidence is therefore urgently needed to inform policymakers, researchers, and stakeholders designing safety net nutrition policies during the current moment and in future crises.

## Methods

### Data

Data were drawn from the publicly available US Census Household Pulse Survey (HPS), a nationally representative serial cross-sectional survey collected weekly to monthly from April 2020 to the present^(25)^. HPS participants are randomly selected by the Census Bureau during each wave to complete an Internet questionnaire, and in this study we used data from waves 13 to 27 (August 19, 2020 to March 29, 2021). The 15 % SNAP benefit increase was implemented on January 1, 2021 (just before wave 22), providing nine waves (18 weeks) of pre-policy and six waves (12 weeks) of post-policy data. For this analysis, we restricted HPS data to these waves to isolate a period during the pandemic with the least number of changes to other safety net programmes and policies that could have differentially affected SNAP recipients versus non-recipients, such as expansion of unemployment insurance or receipt of the Pandemic Electronic Benefit Transfer (P-EBT) program for school children^(7)^. This also restricted the study period to Phases 2.0 and 3.0 of the HPS, two versions of the survey with few differences affecting the variables used in the present analysis^(26)^. We further restricted the sample to respondents who had at least one outcome of interest reported, and who were not missing income and household size (to calculate SNAP eligibility in the next step). We then restricted the sample to those who were SNAP-eligible based on whether their self-reported demographics met federal eligibility criteria (see Supplement). Federal SNAP eligibility is determined using adjusted gross income and household size cut-offs of 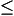 130 % Federal Poverty Limit (FPL). In the USA, this is on average $29 940 a year^(27)^. Lastly, we restricted the sample to those with no missing values for other covariates (see eFigure 1, sample selection flowchart). Complete case analysis (i.e. dropping observations with missing data) is not thought to introduce bias at low levels of missingness like those in the present study^(28–32)^.

### Exposure

In the analysis described below, SNAP-eligible individuals with self-reported receipt of SNAP benefits who were interviewed after implementation of the 15 % benefit increase on January 1, 2021, were considered ‘exposed’. Meanwhile, SNAP recipients interviewed before January 1, 2021 and SNAP-eligible individuals who did not report receipt of the benefit were considered unexposed. Prior work has indicated that self-reported receipt of safety net benefits may be unreliable, which may introduce measurement error due to underreporting of programme receipt^(33–35)^. There are numerous hurdles to linking administratively derived SNAP participation data with health data, and future studies should replicate this analysis if this becomes more feasible. Nevertheless, imputing safety net eligibility as we do here is something that is commonly done in prior work^(18,36,37)^. There was minor state variation in the first date when the 15 % expansion to EA was provided to SNAP recipients, and recent research has highlighted the importance of methods that account for staggered treatment^(38)^. Nevertheless, accounting for staggered timing in this study was not possible as state implementation of expanded benefits occurred in rapid succession; meanwhile, the timing of HPS survey waves (i.e. biweekly or monthly) meant the majority of EA issuance dates occurred between HPS waves. Thus, ‘pre’ and ‘post’ survey waves would have remained the same.

### Outcomes

We examined primary outcomes related to household nutrition and mental health, and secondary outcomes related to financial well-being. We included three nutrition-related outcomes. First was a binary variable for moderate to severe food insufficiency in the last 7 days. Food insufficiency – which is conceptually similar to food insecurity – was designed by the US Department of Agriculture (USDA) to assess rapid changes over time (i.e. within the last 7 d), as opposed to the standard measure of food insecurity which asks about access to food over a longer recall period^(39)^. Further details for this outcome can be found in the Supplement. The second outcome was whether children in the household were often or sometimes not eating enough due to inability to afford food in the last 7 d. The third outcome indicated whether respondents or their household had received free groceries within the last 7 d from a food pantry, church, or other place that helps with free food.

We included two mental health-related outcomes. First, depressive symptoms were measured using the validated two-item Patient Health Questionnaire (PHQ-2) and included as a continuous variable (range 0–6). Second, we included the validated two-item Generalized Anxiety Disorder (GAD-2) scale as a continuous variable (range 0–6)^(40,41)^.

Lastly, we included two binary outcome variables capturing financial hardship to investigate whether the added SNAP benefits offset the burden of other financial stressors. The first variable indicated whether the respondent was currently caught up on rent or mortgage, and the second indicated if they had had difficulty paying household expenses within the last 7 d.

Details on how outcome variables were constructed based on HPS questions are included in the Outcomes section of the Supplement.

### Covariates

We adjusted models for variables that could potentially confound the relationship between the outcomes of interest and exposure to the SNAP benefit increase: gender, race/ethnicity, income, marital status, household size, education, age, and work loss during COVID-19. We included fixed effects (i.e. indicator variables) for state of residence, as state factors may influence both SNAP take-up as well as the outcomes of interest, as well as fixed effects for survey wave to account for secular trends in outcomes that occurred during our study period due to underlying (e.g. pandemic-related) factors that affected all individuals.

### Statistical analysis

#### Primary analysis

We first tabulated descriptive statistics stratified by self-reported SNAP receipt and whether the survey took place before or after the SNAP benefit increase in January 2021.

Next, we estimated the short-term effect of the benefit expansion using difference-in-differences (DID) analysis, a common quasi-experimental technique for policy evaluation that accounts for secular trends^(42)^. Specifically, DID analysis compared federally SNAP-eligible individuals who reported receiving SNAP benefits after January 2021 with federally SNAP-eligible individuals who reported receiving SNAP benefits pre-January 2021. Then, to factor out possible secular trends in outcomes over time, DID analysis ‘differences out’ the pre-post changes observed among a control group of SNAP-eligible individuals who did not report receiving SNAP.

Importantly, DID analysis does not require that the treatment and control groups be similar in all respects, and indeed, there are reasons to think that SNAP-eligible individuals who do not receive benefits differ from actual recipients. Rather, DID analysis involves several assumptions. The first is that pre-post differences in outcomes would have been similar between SNAP recipients and non-recipients in the absence of the benefit increase. This counterfactual scenario cannot be tested; however, we can nevertheless assess whether non-recipients represent a comparable control group by assessing the ‘parallel trends assumption’ of DID, that is, whether trends in outcomes during the pre-period were parallel for recipients and non-recipients (eMethods, eFigure 2, eFigure 3, and eTable 1). Second, DID assumes there are no differential compositional changes between the treatment and control group over time that might affect the outcomes. To evaluate the validity of this assumption, we assessed whether pre-post changes in observed covariates were similar among SNAP recipients and non-recipients (eMethods, eTable 2). A third assumption of DID is the absence of other exposures that differentially influence outcomes for the treatment and control groups at the same time as the exposure of interest, such as other co-occurring policies. As noted above, prior documentation of pandemic-related policymaking during this time suggests no major safety net or other policy changes occurred that would have affected SNAP-eligible recipients differently than eligible non-recipients^(9)^. However, we are never fully able to rule out the existence of any such co-occurring policies, particularly given the dynamic policy landscape in the USA during the pandemic^(9)^. The potential for residual confounding is a limitation of all DID analyses.

Following the standard DID approach, we estimated multivariable regression models in which the primary exposure variable was an interaction term between whether the observation was recorded after the SNAP benefit increased and whether the individual was a SNAP recipient. These models adjusted for the covariates above and included heteroskedasticity-robust standard errors. Linear regressions were used for both binary and continuous outcomes, as is standard in DID, due to differences in the interpretation of interaction terms in non-linear models^(43)^. Coefficients for binary outcomes are therefore interpreted as percentage-point changes in risk. The Supplement includes additional details and equations (eMethods). All tests were two-tailed, and *P*-values less than 0·05 were considered to be statistically significant.

#### Secondary analyses

##### Subgroup analyses

We conducted subgroup analyses to evaluate whether the benefit increase had differential effects among higher-risk subgroups that may be more likely to benefit from the additional resources, including racial/ethnic minorities and the lowest-income individuals, as existing evidence has shown differential effects of SNAP EA by income and race/ethnicity^(44–46)^. Previous findings from published literature have also shown that safety net programmes may have differential effects among various subgroups because of differences in underlying risk factors, take-up, or other contributing factors. Given previously documented racial, ethnic, and sociodemographic disparities in food insecurity, mental health, and financial hardship brought on by the pandemic^(47)^, this study therefore estimated whether effects differed among key subgroups. To do so, we applied the standard DID analysis above and additionally included a triple interaction term between these subgroup variables and the primary exposure variable (eMethods).

##### Sensitivity tests

We conducted additional analyses to test the robustness of results to alternative specifications. First, we restricted the sample to those who were SNAP-eligible based on their state-specific gross annual income limits (ranging from 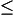 130 % to 200 % FPL), rather than the federal income limits ( 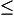 130 % FPL only). While this increases the sample size to those in higher-income categories compared to the federal eligibility threshold, it also introduces potential measurement error because states often additionally define eligibility using Broad Based Categorical Eligibility, which incorporates participation in Temporary Assistance for Needy Families (TANF) and Medicaid^(48)^, and asset limits that are not available in HPS. Therefore, we are only able to use state-specific income guidelines to infer SNAP eligibility in this sensitivity analysis. Nevertheless, this approach has been used in other published work investigating SNAP participation and healthcare utilisation^(49)^.

In a second sensitivity analyses, we used alternate definitions of high and very high food insufficiency. Third, we dichotomised the depressive and anxiety symptom outcomes as binary variables using standard cut-offs of ≥ 3. Additional details on these analyses are in the Supplement (eMethods).

To evaluate concerns around self-reporting of SNAP receipt^(33–35)^, we plotted the proportion of respondents who self-reported SNAP receipt by HPS survey waves (eFigure 4). Next, we conducted a sensitivity analysis in which all SNAP-eligible individuals were assumed to have received SNAP (i.e. were ‘treated’), regardless of self-reported receipt. Those who were ineligible due to reported income above state-level SNAP income cut-offs (but whose income was still < 250 % of the FPL) were considered the ‘control’ group of ‘near eligible’ individuals. This is analogous to an intent-to-treat analysis and overcomes limitations due to misreporting of SNAP receipt^(33–35)^. Methods are further discussed in the Supplement (eMethods).

## Results

### Sample characteristics

The final SNAP-eligible sample included 18 900 SNAP recipients (12 416 before and 6484 after the benefit increase) and 25 577 non-recipients (16 141 before and 9436 after the benefit increase) (Table 1). SNAP recipients in our sample were more likely to be female and Black with lower income and educational attainment than eligible non-recipients. SNAP recipients were also more likely to have lost work during the pandemic. Food insufficiency and mental health problems were higher among SNAP recipients (Table 1). Importantly, DID analysis does not require that characteristics of the treatment and control groups be similar, but rather that trends (i.e. slopes) in outcomes be parallel during the pre-period. Both naïve and event study assessments of pre-parallel trends showed generally parallel trends between treatment and control groups for all outcomes (eFigure 2, eFigure 3, and eTable 1). Further analyses to evaluate these model assumptions were also reassuring and are described in the Supplement ( eMethods, eTable 2).

**Table 1:** Demographic characteristics of study sample, before and after SNAP benefit increase

	SNAP-eligible non-recipients *n* 25 577						SNAP recipients *n* 18 900					
	Pre			Post			Pre			Post		
	*n* 16 141			*n* 9436			*n* 12 416			*n* 6484		
Variables	Percent	Median	IQR	Percent	Median	IQR	Percent	Median	IQR	Percent	Median	IQR
*Demographic characteristics*												
Male	32·7			32·8			18·1			19·8		
Race/ethnicity												
Non-Hispanic White	54·4			53·4			48·5			46·7		
Non-Hispanic Black	11·0			10·0			20·0			20·0		
Hispanic	21·8			23·6			19·9			22·2		
Non-Hispanic Asian	5·6			6·4			2·7			2·7		
Non-Hispanic Other	7·2			6·6			8·9			8·3		
Annual income (US$)												
< $25 000	71·9			72·5			79·1			79·1		
$25 000–$34 999	22·2			21·6			17·1			16·7		
$35 000+	5·9			5·9			3·8			4·2		
High school education or less	31·3			30·9			41·0			41·9		
Age (years)		42·0	31·0–56·0		44·0	32·0–59·0		40·0	33·0–51·0		42·0	35·0–54·0
Household size		4·0	3·0–5·0		4·0	3·0–5·0		4·0	3·0–5·0		4·0	3·0–6·0
Married	36·1			35·5			29·8			30·1		
Work loss during COVID-19	61·8			61·3			67·8			69·3		
*Outcomes*												
Household food insufficient	65·2			61·9			73·0			68·0		
Children not eating because cannot afford food	43·3			43·4			44·6			45·6		
Free groceries/meals	16·6			14·7			30·8			28·4		
Anxiety symptoms (PHQ-2)		2·0	1·0–4·0		2·0	1·0–5·0		3·0	2·0–5·0		3·0	1·0–5·0
Depressive symptoms (GAD-2)		2·0	0·0–4·0		2·0	0·0–4·0		2·0	1·0–4·0		2·0	1·0–4·0
Somewhat/very difficult to pay expenses	58·7			58·4			75·7			72·8		
Caught up on rent/mortgage	77·9			77·0			69·0			67·3		

SNAP, Supplemental Nutrition Assistance Program; PHQ-2, Patient Health Questionnaire 2-item scale; GAD-2, Generalized Anxiety Disorder 2-item scale.

*n* 44 477. Data were drawn from the US Census Bureau Household Pulse Survey, August 2020 to March 2021 waves.

### Effects of 15 % SNAP benefit increase

**Fig. 3:**
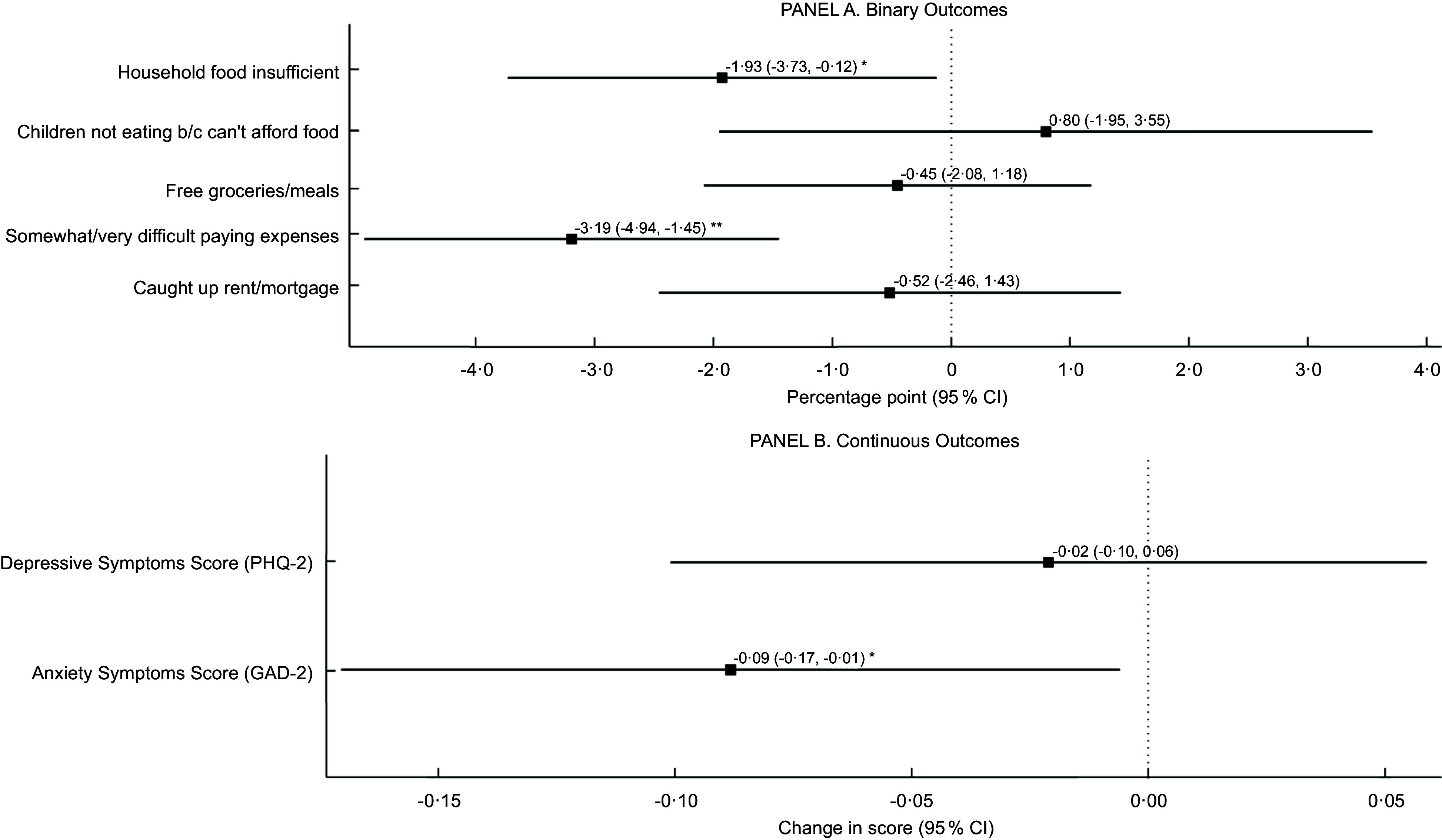
Effect of SNAP benefit increase on health and financial well-being. **P < 0·01, *P < 0·05. *n* 44 477. SNAP, Supplemental Nutrition Assistance Program; GAD-2, Generalized Anxiety Disorder 2-item scale; PHQ-2, Patient Health Questionnaire 2-item scale; b/c, because. Data were drawn from the US Census Bureau Household Pulse Survey, August 2020 to March 2021 waves. Estimates represent the coefficient on the interaction term from difference-in-differences models adjusted for gender, age, marital status, income, household size, race/ethnicity, education, and work loss during COVID-19, as well as fixed effects for state and survey week, with robust standard errors

The SNAP benefit increase was associated with decreased food insufficiency (–1·9 percentage points (pp); 95 % CI –3·7, –0·1), decreased anxiety symptoms (–0·09 points; 95 % CI –0·2, –0·01) and decreased difficulty paying for other household expenses (–3·2 pp; 95 % CI –4·9, –1·5) (Fig. 3). We were unable to reject the null hypothesis of no improvements in other outcomes, including child food insufficiency, receiving free meals, depressive symptoms, or being caught up on rent/mortgage.

### Secondary analyses

#### Subgroup analyses

There was a larger increase in households who received free groceries or meals in the last 7 d among Hispanic SNAP-enrolled households (5·7 pp; 95 % CI 1·2, 11·0) and Asian households (13·0 pp; 95 % CI 3·5, 22·6) compared with White households. There were also larger improvements in Asian SNAP-enrolled households being caught up on rent/mortgage (12·3 pp; 95 % CI 0·9, 23·8). We found no other subgroup differences by race/ethnicity or income (eTable 3).

#### Sensitivity analyses

When using state-specific SNAP income eligibility criteria, the sample size roughly doubled from that of the primary analysis (*n* 96 768). Analyses to evaluate model assumptions were reassuring. Results were similar to the primary analysis: the SNAP benefit increase was associated with similar decreases in food insufficiency (–1·6 pp; 95 % CI –2·9, –0·3), difficulty paying for household expenses (–1·9 pp; 95 % CI, –3·1, –0·6), and anxiety symptoms (–0·07 points; 95 % CI –0·1, –0·01) compared with the primary analysis (eTable 4).

In sensitivity analyses examining binary (rather than continuous) versions of the food insufficiency and mental health outcomes, we were unable to reject the null hypothesis of no association (eTable 5). Nevertheless, the direction of association was consistent with the primary analysis, suggesting that these analyses may have been underpowered when converting the continuous scales to binary variables, or alternatively, that the additional benefits were enough to reduce anxiety symptoms, but not enough to move symptoms across PHQ-2 score thresholds indicative of high risk of depression or anxiety^(40,41)^.

Results for the intent-to-treat analysis can be found in eTable 6. Due to HPS reporting income ranges by category, rather than a continuous measure, observations whose reported income category range overlapped with a SNAP eligibility income threshold for household size were not included in this analysis. Quantitative assessments of parallel trends showed violations for outcomes child food insufficiency, GAD-2, difficulty with household expenses, and caught up on rent/mortgage; thus, findings for this sensitivity analysis should be interpreted cautiously due to potential measurement error and the validity of the proposed control group.

## Discussion

Since the onset of the COVID-19 pandemic, SNAP has been at the forefront of America’s safety net response, expanding monthly allotments, online food purchasing, and application waivers to counteract pandemic-related economic hardship^(9,11)^. Using a large serial cross-sectional national dataset and quasi-experimental analysis, this study examined the effects of the pandemic-era 15 % SNAP increase on food insufficiency, mental health, and financial well-being. We found that the benefit increase was associated with not only a reduction in food insufficiency, consistent with one prior study using the same dataset^(17)^, but also a reduction in anxiety symptoms and difficulties paying for other household expenses. Additionally, findings were robust to sensitivity analyses using state-level income eligibility criteria.

About one in eight Americans receive SNAP benefits^(50)^, and the reduction in the prevalence of food insufficiency and difficulty paying for household expenses represent a meaningful change in the distribution at the population level. The observed 1·9 pp reduction in food insufficiency represents a 2·6 % reduction from baseline food insufficiency levels (73·0 %) among SNAP recipients in our sample. These findings are similar in magnitude to recent pandemic-era studies using HPS data which found at least 3 pp increase in food insufficiency after EA expiration^(45,46,51)^, and others which report a 3·7 pp reduction in food insufficiency after the pandemic-related Child Tax Credit (CTC) expansion – which provided substantial income benefits to families with children from July to December, 2021^(52)^. Although we did not find significant improvements in child food security, past research suggests that food insufficiency/insecurity among children is low because parents are the first to forgo food to be able to provide food for their children^(53)^.

Food insufficiency and financial insecurity are known risk factors of poor mental health^(1,2)^, which supports our findings of a modest reduction in anxiety symptoms among SNAP recipients after the benefit increase. The 0·09-point reduction in the PHQ-2 score represents a 3·3 % reduction from baseline anxiety levels (2·7 points) among SNAP recipients in our sample. Prior studies on other economic support programmes, such as the Earned Income Tax Credit and CTC, have shown larger improvements in mental health after benefit expansions^(54,55)^. One study in particular using HPS data found that the pandemic-era CTC expansion reduced anxiety levels among recipients by 3·4 pp and depression by 1·7 pp^(55)^. It is possible that the smaller effect size for anxiety observed in the present study was due to the nature of the food-specific benefit or the smaller monetary size of the benefit increase, while the CTC was larger and allowed for more spending flexibility. Although small effect sizes are indeed less clinically meaningful at the individual level, they represent changes in the distribution at the population level, which can be impactful for public health^(31)^.

SNAP benefits are issued once per month, and pre-2020 expenditure data shows that nearly 80 per cent of SNAP benefits were redeemed within 2 weeks of receipt, leaving enrollees with less support for the second half of the month^(19)^. The 3·2 pp reduction in difficulty with household expenses, which represents a 4·2 % reduction from baseline (75·7 %) among SNAP recipients, underscores the potential for SNAP benefits to significantly affect household financial well-being. It is possible that the 15 % SNAP increase may have improved food availability towards the end of the month, in turn improving food sufficiency and anxiety around having enough food and other resources. Prior work has found that increasing SNAP benefits not only increases expenditure on food but also increases expenditures on other household items, further evidence that SNAP benefits free up households’ funds for other purchases^(19,21,56)^. Less is known of differential effects of pandemic-era SNAP modifications by cost-of-living status, although prior work suggests that benefits are even less sufficient at meeting recipients’ needs in high-cost areas^(56–59)^. The present findings highlight the need for further research to determine if this 15 % benefit boost succeeded in offsetting household expenses even in high-cost areas, particularly during this period of persistent high inflation^(23)^.

We also found differences in effect estimates for some outcomes among key subgroups. For example, Hispanic and Asian respondents were more likely than non-Hispanic White respondents to receive free groceries/meals after the SNAP increase. Although the sources of free meals are unknown (e.g. food bank, family/friend) due to data limitations, our findings suggest that perhaps this SNAP increase was not sufficient for certain subgroups during the pandemic due to pre-existing racial and ethnic disparities that were further exacerbated during this time period^(7,60)^. Or perhaps, stronger community networks and/or awareness of community resources among these groups led to higher use of food banks during this period^(61)^. It is also possible that other ongoing factors – such as anti-Asian racism – may have outweighed any benefits from the temporary income boost or caused Asian individuals to fear further stigma from enrolling in a safety net programme such as SNAP and turned instead to community organisations for assistance^(5,60,62,63)^. Another alternate explanation is that Hispanic and Asian individuals were more likely to have an immigration status that rendered them ineligible for SNAP^(64,65)^; however, data limitations in HPS precluded the inclusion of immigration status (or a proxy variable such as country of birth) in the present analyses. Asian households also faced less difficulty paying rent/mortgage compared with non-Hispanic White households after the SNAP expansion. Given that we did not see consistent patterns across subgroups, these findings should be interpreted cautiously in light of multiple hypothesis testing and warrant future investigation.

This study has several strengths. We used a large serial cross-sectional diverse national dataset and a rigorous quasi-experimental study design to assess associations between a major safety net expansion during the pandemic and health outcomes^(42)^. Food insecurity and mental health remain among the top public health areas of concern and warrant increased attention owing to exacerbation from the COVID-19 pandemic^(1,2,5,6)^. This study also has several limitations. First, HPS suffers from a high rate of non-response, as with many other national surveys; results therefore may not generalise to those not included in this study^(26)^. The Census Bureau considers the HPS an experimental data product, as it has not gone through the same types of review and testing as other Census products^(26)^. Second, HPS is a repeated cross-sectional survey, so we were unable to observe changes in specific individuals’ outcomes. Covariates, outcomes, and SNAP receipt were self-reported and may suffer from standard reporting biases, for example, due to misreporting of SNAP and other safety net programme participation^(34,35)^. However, we found stable SNAP receipt across all survey waves in the study period (eFigure 4). Another source of potential bias may exist from increased enrolment in SNAP as a result of the increased benefits during the pandemic^(66)^. Additionally, because HPS reported annual income by category and did not include any enrolment data for safety net programmes such as Medicaid or TANF that are used in many states to determine SNAP eligibility, some individuals with incomes near the eligibility cut-offs were dropped from our sample to avoid possible misclassification. Therefore, results may not be generalisable to those right around the income cut-offs. Lastly, as with any DID analysis, there may be residual confounding based on contemporaneous policy changes or other exposures that differentially affected treatment and control groups. To address this issue, we restricted our study period to a narrow window of 12 weeks after the benefit increase to avoid other policy changes.

## Conclusion

Despite programme expansions, SNAP participation and enrolment remained stagnant through 2020 and into 2021 among low-income groups and declined among families with children^(67,68)^. Although a permanent SNAP benefit increase of about 21 % was implemented in October 2021 through the Thrifty Food Plan to adjust for cost of living^(12,44)^, debates continue over whether pandemic-era safety net programme expansions, including SNAP benefit increases, were sufficient in addressing the country’s health needs. Many of the core anti-poverty programmes enacted during the pandemic have expired, with some states ending all pandemic-era SNAP EA as early as April 2021 through March 2023^(69)^. Additionally, inflation continues to drive high food prices and food insecurity^(23)^. Recent research shows that the expiration of SNAP EA led to increased food insufficiency and financial insecurity despite Thrifty Food Plan increases^(45,46,51)^. Timely evidence regarding the potential health effects of recent SNAP programme modifications is urgently needed to inform the further expansion of anti-poverty programmes, particularly given the persistence of socio-economic consequences of the COVID-19 pandemic^(18)^. Evidence from this study builds on a growing knowledge base of the effects of the pandemic-era safety net response and informs policymaking to address the health and social consequences of economic downturns.

## Supporting information

Jackson et al. supplementary material 1 Jackson et al. supplementary material

Jackson et al. supplementary material 2 Jackson et al. supplementary material

## Supplementary material

For supplementary material accompanying this paper visit https://doi.org/10.1017/S1368980024001447
