# Supplementary material for: The association of increased SNAP benefits during COVID-19 with food insufficiency and anxiety among US adults: a quasi-experimental study
Source: Public Health Nutr. 2024 Sep 27;27(1):e186. doi: 10.1017/S1368980024001447 (PMC11504391; doi:10.1017/S1368980024001447)
Supplement: Jackson et al. supplementary material 2 — Jackson et al. supplementary material [file S1368980024001447sup002.pdf]

SUPPLEMENT

Supplemental Methods

Data

Two phases of the Household Pulse Survey (HPS) were included in the present analysis, Phase 2.0 and Phase 3.0. To ensure there was no differential missingness between key analytic variables between survey phases, we calculated the percent missingness for these variables. Specifically, between Phases 2.0 and 3.0, the average rates of missingness differed by <1% for model covariates, and by <0.1% for all outcomes. Child food insufficiency differed by <1% between survey phases.(1)

Supplemental Nutrition Assistance Program (SNAP) Eligibility

To be federally eligible for SNAP benefits, participants must report annual gross income at or below 130% of the Federal Poverty Level (FPL).(2) Alaska and Hawaii are exceptions with higher income limits due to higher cost of living; this was accounted for in our analyses. HPS reports income in categories, rather than as a continuous measure, and therefore, the threshold for 130% of the FPL fell within one of these categories in some cases. To avoid including ineligible individuals in our sample, we dropped individuals falling into categories that included an income cutoff threshold.

Outcomes

To assess food insufficiency in the HPS, respondents were asked – "*In the last 7 days, which of these statements best describes the food eaten in your household?*" Respondents could answer: 1) enough of the kinds of food (I/we) wanted to eat, 2) enough but not always the kinds of food

(I/we) wanted to eat, 3) sometimes not enough to eat, 4) often not enough to eat. In our primary analysis we assessed what is considered "marginal food insufficiency" using the household food sufficiency scale. Marginal food insufficiency is defined by the U.S. Department of Agriculture (USDA) as a household that reports having enough to eat but not always the kinds of food they wanted to eat in the last 7 days.[3] Therefore, HPS respondents who selected options 2 through 4 were classified as experiencing food insufficiency for the purpose this analysis.

The second outcome related to food insufficiency was whether children in the household were often or sometimes not eating enough due to inability to afford food. The sample size for this outcome of interest was 34,279 respondents with children. For this question, respondents were asked to indicate whether the statement – "*The children were not eating enough because we just couldn't afford enough food*." – was 1) often true, 2) sometimes true, or 3) never true in the last 7 days for the children living in the household who are under 18 years old. Respondents who answered either 1 or 2 were combined as "Yes" for this binary variable.

For the third nutrition-related outcome variable, respondents were asked in the HPS – "*During the last 7 days, did you or anyone in your household get free groceries or a free meal*?" – to which they could respond 1) Yes or 2) No.

In HPS, the two-item Patient Health Questionnaire (PHQ-2) assessing depressive symptoms was comprised of two questions; 1) "*Over the last 7 days, how often have you been bothered by…having little interest or pleasure in doing thigs?*", and 2) "*Over the last 7 days, how often have you been bothered by feeling down, depressed, or hopeless?*" The HPS also asked

respondents two questions for the two-item Generalized Anxiety Disorder (GAD-2) scale; 1) "*Over the last 7 days, how often have you been bothered by the following problems – not being able to stop or control worrying?*" and 2) "*Over the last 7 days, how often have you been bothered by the following problems - feeling nervous anxious, or on edge?*". For each of these questions, respondents could answer from the following: 1) Not at all, 2) Several days, 3) More than half of the days, and 4) Nearly every day. For each two-item score, responses were summed across the two questions to generate a continuous score between 0 and 6. Scores ≥3 indicate high risk of depression or anxiety.[4, 5]

For the binary variable representing somewhat/very difficult to pay expenses, respondents were asked in the HPS survey: "*In the last 7 days, how difficult has it been for your household to pay for usual household expenses, including but not limited to food, rent or mortgage, car payments, medical expenses, student loans and so on?*" Respondents could choose from the following choices: 1) Not at all difficult, 2) A little difficult, 3) Somewhat difficult, 4) Very difficult. Respondents who chose options 3-4 were coded as having difficulty with expenses in this binary variable. Lastly, for the binary variable representing respondents currently caught up on rent/mortgage was assessed using the two following questions in the HPS: 1) "*Is this household currently caught up on rent payments?*" or 2) "*Is this household currently caught up on mortgage payments?*"

Difference-in-differences Analysis

The equation for the difference-in-differences (DID) model, in which the analysis is at the individual level, is:

$$Y = \alpha + \beta_1 Post \times SNAP + \beta_2 Post + \beta_3 SNAP + \beta_4 Covars + \beta_5 Week + \beta_6 State + \varepsilon$$

Y represents an outcome of interest. The variable $Post$ indicates whether the observation was recorded before or after the SNAP benefit increase on January 1, 2021. The variable $SNAP$ indicates whether the individual self-reported receipt of SNAP benefits. $Covars$ represents individual-level covariates described in the main text, $Week$ and $State$ represent fixed effects (i.e., indicator variables) for survey week and state of residence, respectively, and ε represents heteroskedasticity-robust standard errors.

Model Assumptions

We qualitatively assessed the parallel trends assumption of DID by plotting outcome trends for SNAP-eligible recipients versus non-recipients during the pre-period (**eFigure 2**). For most outcomes, the graphs demonstrated roughly parallel trends, although trends for child food insufficiency and being caught up on rent/mortgage were noisy, so results for these outcomes should be interpreted cautiously. We additionally assessed parallel trends graphically by plotting event study plots, showing the average treatment effect on the treated (ATT) in each wave (**eFigure 3).** An event study is a more flexible version of traditional DID, in which the primary exposure of interest is an interaction term between a continuous survey wave variable and the treatment group indicator, and accounts for variability.[(6)] Models were constructed using the Stata package *eventedd*,[(6)] and were adjusted for gender, age, marital status, income, household size, race/ethnicity, education, and work loss during COVID-19, as well as fixed effects for treatment assignment (i.e., SNAP receipt), state, and survey week, with robust standard errors. Overall, results from event studies matched our naïve assessment of parallel trends in **eFigure 2** for most outcomes, showing roughly parallel trends between exposed and unexposed groups

during the pre-period. Additional studies, also leveraging HPS data, have applied similar event study methods to investigate the health effects of the Child Tax Credit expansion.[7, 8] Lastly, we performed a quantitative evaluation of the validity of the parallel trends assumption by restricting the data to the pre-period and regressing each outcome on an interaction term between SNAP-receipt versus non-receipt and a continuous variable for week of survey. In these tests apart from food insecurity, all coefficients were small and not statistically significantly different from zero, which suggests that trends were parallel (**eTable 1**). The coefficient for food insecurity, although statistically significant, was small (<0.01) and does not represent a substantive difference between groups. Event study results for food insufficiency only showed significant differences for two of the nine pre-period waves, which is reassuring. Nevertheless, results for this outcome should be interpreted cautiously.

A second DID assumption is that there are no differential compositional changes in the treatment and control groups during the study period. To evaluate this assumption, we regressed each sociodemographic characteristic as the dependent variable in a model in which the primary exposures were the same as the main model: SNAP receipt vs non-receipt, whether the survey took place after the SNAP benefit increase, and an interaction between the two. A null result for the interaction term in these regressions would suggest that there were no differential pre-post changes in composition among SNAP recipients and non-recipients. We did not find statistically significant differences for any covariates of interest except for the variable representing any work loss during the pandemic (**eTable 2**). Nevertheless, we controlled for all these variables in our primary analyses to account for potential confounding, but cannot rule out differences in unmeasured confounders, a limitation of any DID analysis. Lastly, although **Figure 1** shows that

SNAP modified eligibility for work study-eligible college students shortly following the 15% increase, this modification was unlikely to differentially affect our sample and is therefore not a large concern.

Subgroup Analyses

Coefficients for subgroup analyses are derived from models in which the primary exposure is a triple interaction term between an indicator for whether the interview occurred after (versus before) the SNAP increase, a binary variable representing SNAP receipt, and a categorical or binary variable for race/ethnicity or income subgroup. The equation for the model is:

$$Y = \alpha + \beta_1 Subgroup \times Post \times SNAP + \beta_2 Subgroup \times Post + \beta_3 Subgroup \times SNAP + \beta_4 Post \times SNAP + \beta_5 Subgroup + \beta_6 Post + \beta_7 SNAP + \beta_8 Covars + \beta_9 Week + \beta_{10} State + \varepsilon$$

Y represents the outcome of interest. The variable $Subgroup$ indicates the racial/ethnic or income subgroup category. The variable $Post$ indicates whether the observation was recorded before or after the SNAP benefit increase on January 1, 2021. The variable $SNAP$ indicates whether the individual self-reported receipt of SNAP benefits. $Covars$ represents individual-level covariates described in the main text, $Week$ and $State$ represent fixed effects for week of HPS survey and state of residence, respectively, and ε is the error term. The coefficient of interest is β_1, on the triple interaction term, representing the effect of the 15% SNAP increase on recipients among a given subgroup category compared to the reference group. Results for subgroup analyses can be found in **eTable 3**.

Sensitivity Analyses

Results for the sensitivity analysis using state-level eligibility criteria are reported in **eTable 4**.

We also conducted sensitivity analyses using more severe food insufficiency scores: 1) high or very high food sufficiency defined as a household not having enough to eat sometimes-to-often in the last 7 days and 2) very high food sufficiency only defined as a household not having enough to eat often in the last 7 days.[3, 9] Results for these sensitivity analyses are found in **eTable 5**.

We also conducted another sensitivity analysis comparing SNAP-eligible individuals before versus after January 2021, while "differencing out" the pre-post changes observed among SNAP-ineligible individuals (i.e., "control group"). To generate a more robust control group, this analysis further restricted the study sample to observations "just above" state-level income eligibility cutoffs but no higher than 250% of the FPL (N=230,953). This method is analogous to an intent-to-treat approach and overcomes limitations due to misreporting of SNAP.[10-12] Similar methods have been applied previously to investigate the effects of SNAP program changes on health and healthcare utilization [13, 14] Nevertheless, due to the fact that HPS reports income ranges by category, rather than a continuous measure, observations whose reported income category range overlapped with a SNAP eligibility income threshold for household size were not included in this analysis, leaving out many observations who might otherwise be "just above" an income threshold. Further, although event studies showed generally parallel trends for most outcomes, our quantitative assessment showed violations of parallel trends for the outcomes child food insufficiency, GAD-2, difficulty with household expenses, and caught up on rent/mortgage. Thus, results from this intent-to-treat analysis should be interpreted cautiously

due to potential measurement error and the validity of the proposed control group. Results are found in **eTable 6**.

SUPPLEMENTAL REFERENCES

1. United States Census Bureau (2021) *2021 Household Pulse Survey User Notes*. Household Pulse Survey Technical Documentation. Washington D.C.: United States Census Bureau.
2. U.S. Department of Agriculture Food and Nutrition Service (2022) SNAP Eligibility. https://www.fns.usda.gov/snap/recipient/eligibility (accessed July 11 2022)
3. U.S. Department of Agriculture Economic Research Service (2022) Food Security in the U.S. https://www.ers.usda.gov/topics/food-nutrition-assistance/food-security-in-the-u-s/measurement/ (accessed July 14 2022)
4. Sapra A, Bhandari P, Sharma S *et al.* (2020) Using Generalized Anxiety Disorder-2 (GAD-2) and GAD-7 in a Primary Care Setting. *Cureus* 12, e8224-e8224.
5. Kroenke K, Spitzer RL Williams JB (2003) The Patient Health Questionnaire-2: validity of a two-item depression screener. *Med Care* 41, 1284-1292.
6. Clarke D & Tapia-Schythe K (2021) Implementing the panel event study. *The Stata Journal* 21, 853-884.
7. Batra A, Jackson K Hamad R (2023) Effects Of The 2021 Expanded Child Tax Credit On Adults' Mental Health: A Quasi-Experimental Study. *Health Affair* 42, 74-82.
8. Shafer PR, Gutiérrez KM, Ettinger de Cuba S *et al.* (2022) Association of the Implementation of Child Tax Credit Advance Payments With Food Insufficiency in US Households. *JAMA Network Open* 5, e2143296-e2143296.
9. Bryant A & Follett L (2022) Hunger relief: A natural experiment from additional SNAP benefits during the COVID-19 pandemic. *Lancet Reg Health Am* 10, 100224.
10. Celhay PA, Meyer BD Mittag N (2021) *Errors in reporting and imputation of government benefits and their implications*. National Bureau of Economic Research.
11. Rothbaum J, Fox L Shantz K (2023) *Fixing Errors in a SNAP: Addressing SNAP Under-reporting to Evaluate Poverty*. The U.S. Census Bureau.
12. Giefer KG, King MD Roth VL (2022) *SNAP Receipt in SIPP: Using Administrative Records to Evaluate Data Quality*. SEHSD Working Paper. U.S. Census Bureau.
13. Nord M & Prell M (2011) Food Security of SNAP Recipients Improved Following the 2009 Stimulus Package. https://www.ers.usda.gov/amber-waves/2011/june/food-security-of-snap/ (accessed August 1 2022)
14. Morrissey TW & Miller DP (2020) Supplemental Nutrition Assistance Program Participation Improves Children's Health Care Use: An Analysis of the American Recovery and Reinvestment Act's Natural Experiment. *Acad Pediatr* 20, 863-870.

eFigure 1. Sample size flow

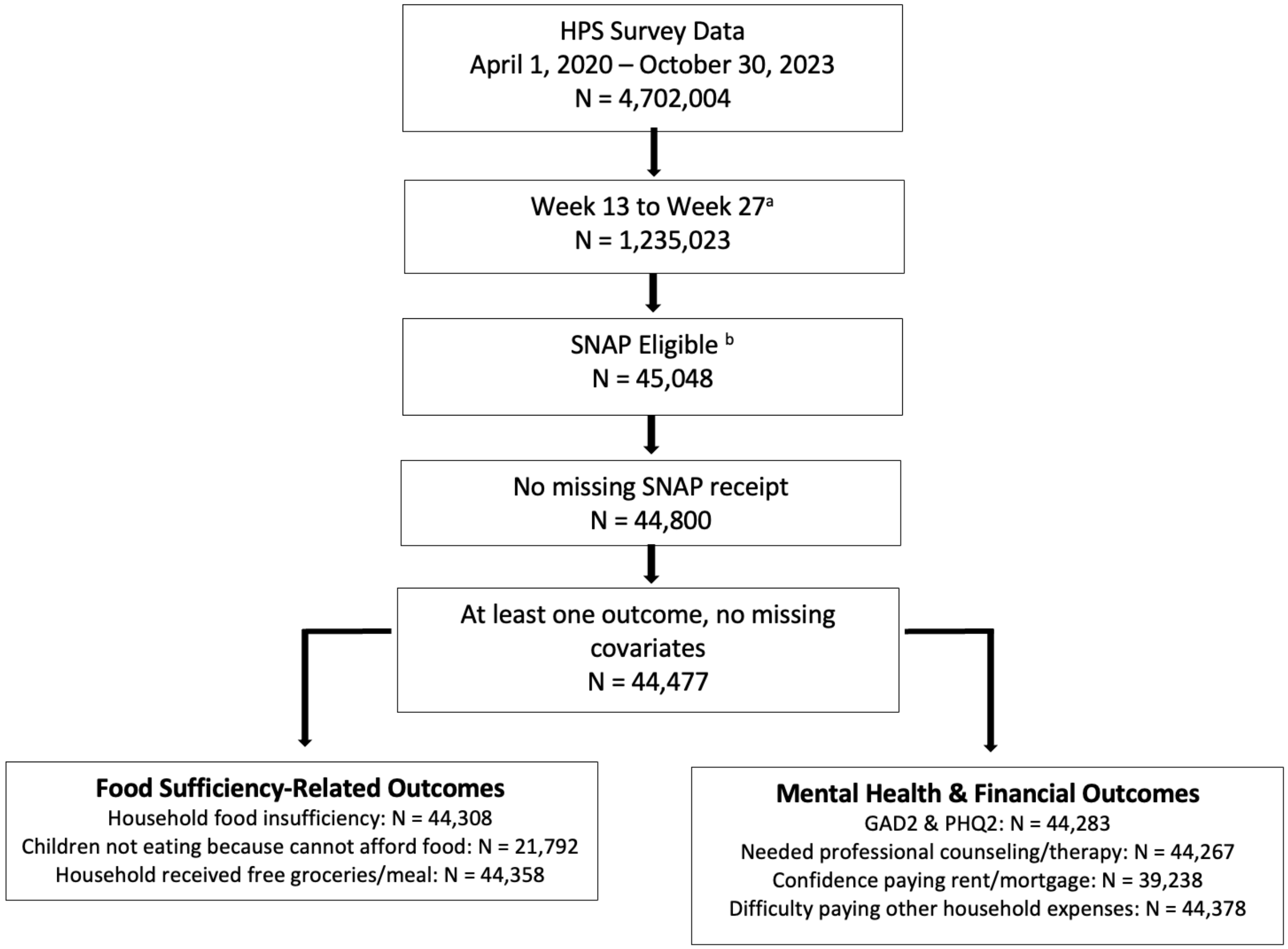

Note: Abbreviations: Household Pulse Survey (HPS); Supplemental Nutrition Assistance Program (SNAP)
Data drawn from the U.S. Census Bureau Household Pulse Survey. The final sample size was different for each outcome based on survey skip logic and/or differential missingness across outcomes of interest.
[a] Date range represents August 19, 2020 to March 29, 2021.
[b] Eligibility determined based on whether self-reported demographics fell within federal SNAP income eligibility guidelines.

eFigure 2. Qualitative evaluation of parallel trends assumption

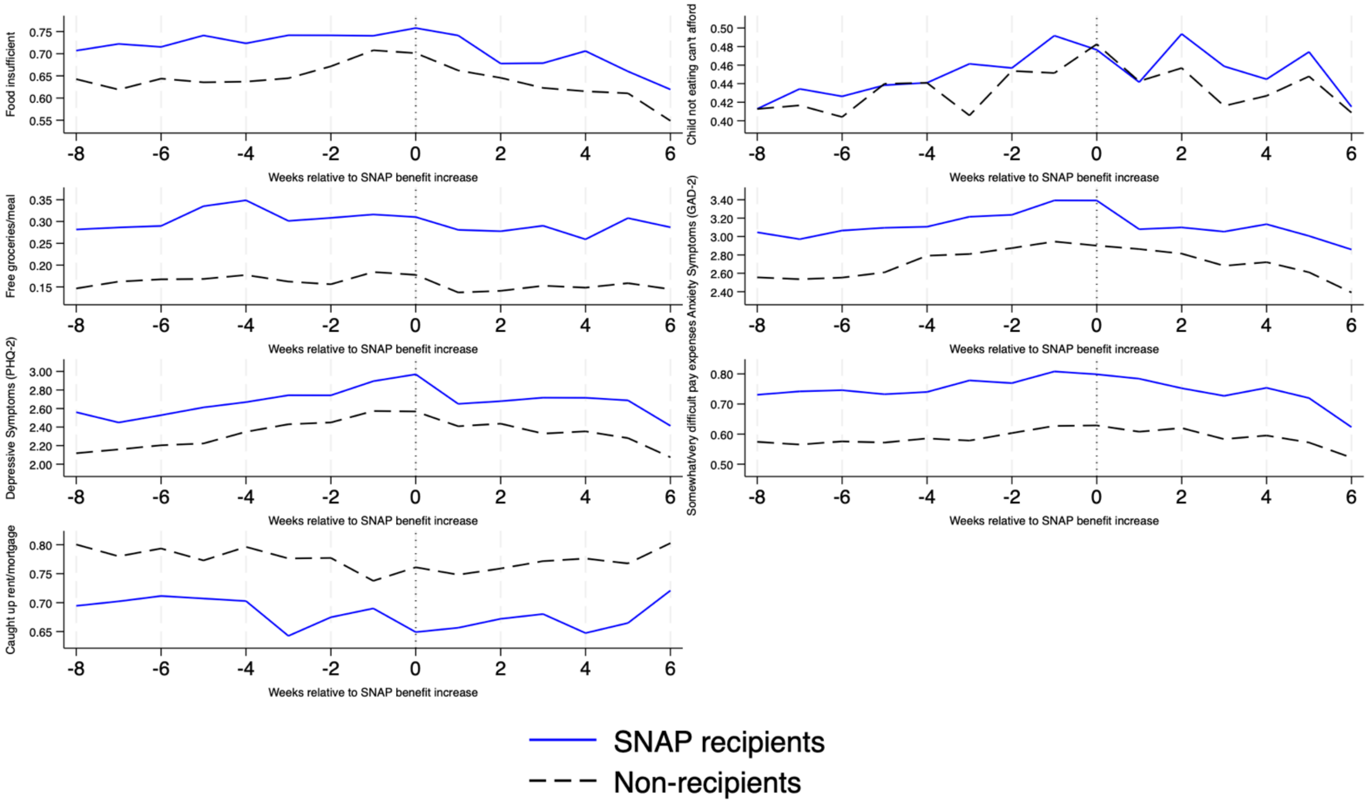

Note: Data drawn from the U.S. Census Bureau Household Pulse Survey, Aug 2020 to Mar 2021 waves. Estimates represent the unadjusted outcome trends over survey wave.
Abbreviations: Generalized Anxiety Disorder 2-item scale (GAD-2); Patient Health Questionnaire 2-item scale (PHQ-2); SNAP; Supplemental Nutrition Assistance Program

eFigure 3. Event study plots

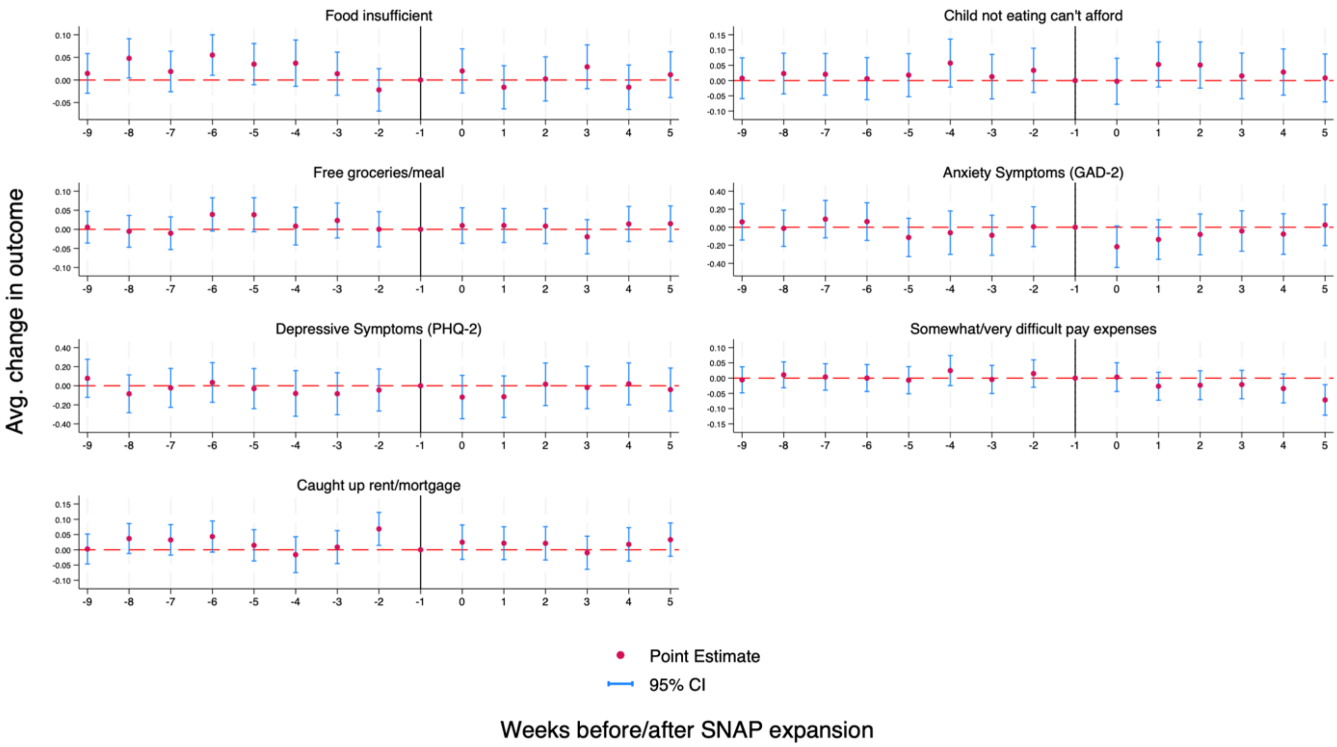

Note: Abbreviations: GAD-2, Generalized Anxiety Disorder 2-item scale; PHQ-2, Patient Health Questionnaire 2-item scale, SNAP; Supplemental Nutrition Assistance Program.
Data drawn from the U.S. Census Bureau Household Pulse Survey, Aug 2020 to Mar 2021 waves. Values along the X axis represent the number of waves relative to the SNAP expansion. Coefficients are derived from event study models in which the primary exposure is interactions between survey wave indicators and the interactions between them and the treatment group indicator (SNAP receipt). Models were adjusted for gender, age, marital status, income, household size, race/ethnicity, education, and work loss during COVID-19, as well as fixed effects for state and survey week, with robust standard errors.

eFigure 4. Self-reported SNAP enrollment by survey wave

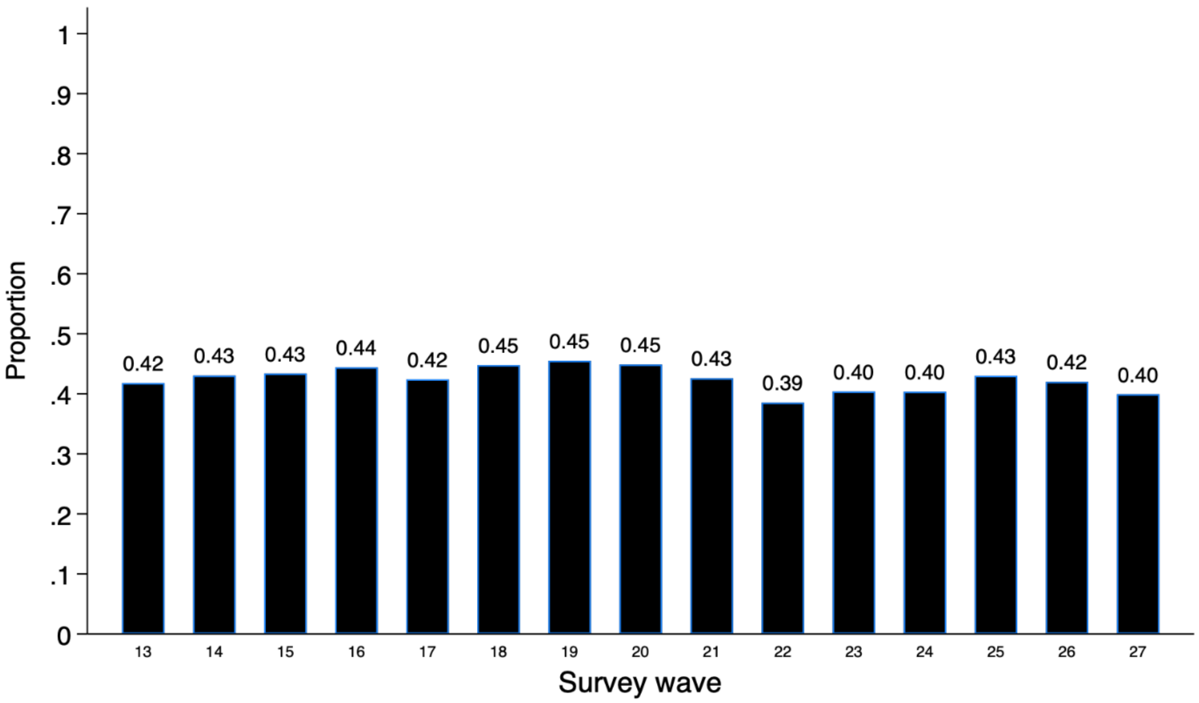

Note: Abbreviations: Supplemental Nutrition Assistance Program (SNAP).
Data drawn from the U.S. Census Bureau Household Pulse Survey, Aug 2020 to Mar 2021 waves. Values along the X axis represent the survey waves.

eTable 1. Quantitative evaluation of parallel trends assumption by outcome

	**Nutrition-related outcomes**			**Mental Health Outcomes**		**Secondary Outcomes**	
	**Household food insufficient**	**Children not eating b/c can't afford food**	**Free groceries/meals**	**Anxiety Symptoms Score (GAD-2)**	**Depressive Symptoms Score (PHQ-2)**	**Somewhat/very difficult paying expenses**	**Caught up rent/ mortgage**
**Coefficient**	-0.005*	0.001	0.001	-0.01	-0.007	0.001	-0.0001
**[95% CI]**	[-0.009, -0.001]	[-0.006, 0.007]	[-0.003, 0.005]	[-0.03, 0.007]	[-0.03, 0.01]	[-0.003, 0.004]	[-0.005, 0.004]
**Observations**	28,440	14,534	28,482	28,449	28,432	28,523	22,148

Note: ** $p < 0.01$, * $p < 0.05$
Abbreviations: GAD-2, Generalized Anxiety Disorder 2-item scale; PHQ-2, Patient Health Questionnaire 2-item scale, SNAP; Supplemental Nutrition Assistance Program
Data drawn from the U.S. Census Bureau Household Pulse Survey, Aug 2020 to Mar 2021 waves. For the purposes of this analysis, the data set was restricted to the pre-period (before January 1, 2021). Coefficients are derived from models in which the primary exposure is an interaction term between a binary variable representing observations who received SNAP (versus non-recipients) and a continuous variable for week of survey.

eTable 2. Evaluation of differential compositional changes in treatment and control groups

**Variables**	**Coefficient**	**[95% CI]**
Male	0.016	[-0.002, 0.03]
Race	0.002	[-0.012, 0.016]
White	0.002	[-0.02, 0.02]
Black	0.007	[-0.006, 0.02]
Hispanic	-0.002	[-0.02, 0.02]
Asian	-0.005	[-0.01, 0.003]
Other	0.001	[-0.01, 0.01]
Annual Income		
<$25,000	-0.008	[-0.03, 0.009]
$25,000-$34,999	0.003	[0.01, 0.02]
$35,000+	0.005	[-0.003, 0.01]
High school degree or below	0.01	[-0.006, 0.03]
Age (years)	0.2	[-0.8, 0.4]
Household size	0.004	[-0.07, 0.07]
Married	0.01	[-0.009, 0.03]
Any work loss during COVID-19	0.02*	[0.000, 0.04]

Note: **p < 0.01, *p<0.05. N = 40,477. Data drawn from the U.S. Census Bureau Household Pulse Survey, Aug 2020 to Mar 2021 waves.
Abbreviations: Supplemental Nutrition Assistance Program (SNAP).
Coefficients are derived from models in which the primary exposure is an interaction term between a binary variable indicating SNAP receipt and an indicator for whether the interview occurred after (versus before) the 15% SNAP benefit expansion. The models examine whether differential compositional differences exist in the demographic characteristics of SNAP recipients versus non-recipients. A null result would indicate that there are no differential compositional changes in the treatment and control groups over time for a given covariate.

eTable 3. Racial/ethnic and income subgroup differences in the effects of the 15% SNAP benefit increase

	Nutrition-related outcomes			Mental Health Outcomes		Secondary Outcomes	
**Subgroup**	**Household food insufficient**	**Children not eating b/c can't afford food**	**Free groceries/meals**	**Anxiety Symptoms Score (GAD-2)**	**Depressive Symptoms Score (PHQ-2)**	**Somewhat/very difficult paying expenses**	**Caught up rent/mortgage**
**Race/Ethnicity (ref: White)**							
Black	3.4	4.2	3.4	0.06	0.08	2.4	-3.2
[95% CI]	[-1.9, 8.8]	[-3.8, 12.3]	[-1.3, 8.0]	[-0.2, 0.3]	[-0.2, 0.3]	[-2.8, 7.5]	[-9.2, 2.8]
Hispanic	-4.5	2.6	5.7**	0.06	0.05	1.3	0.4
[95% CI]	[-9.1, 1.0]	[-4.2, 9.5]	[1.2, 11.0]	[-0.1, 0.3]	[-0.1, 0.3]	[-3.1, 5.8]	[-4.5, 5.4]
Asian	-5.2	-1.4	13.0**	0.02	0.1	1.5	12.3**
[95% CI]	[-15.8, 5.3]	[-30.8, 2.9]	[3.5, 22.6]	[-0.4, 0.4]	[-0.3, 0.5]	[-8.5, 11.5]	[0.9, 23.8]
Other	-5.1	5.1	1.1	0.01	0.01	-0.3	1.2
[95% CI]	[-11.8, 1.7]	[-5.4, 15.6]	[-7.4, 5.3]	[-0.2, 0.4]	[-0.3, 0.3]	[-6.8, 6.2]	[-6.5, 9.0]
**Annual Income (ref: < $25,000)**							
$25,000-$34,999	0.7	-4.1	-1.2	0.1	0.1	-1.3	2.1
[95% CI]	[-4.0, 5.4]	[-10.8, 2.5]	[-3.2, 5.5]	[-0.1, 0.3]	[-0.1, 0.3]	[-5.9, 3.3]	[-2.9, 7.2]
$35,000+	-5.2	-5.3	-2.5	0.3	0.4	-2.2	-2.1
[95% CI]	[-14.0, 4.3]	[-17.2, 6.6]	[-10.9, 5.9]	[-0.04, 0.7]	[-0.005, 0.7]	[-10.7, 6.8]	[-11.1, 6.9]

Note: **p < 0.01, *p<0.05.

Abbreviations: GAD-2, Generalized Anxiety Disorder 2-item scale; PHQ-2, Patient Health Questionnaire 2-item scale, SNAP; Supplemental Nutrition Assistance Program

Data drawn from the U.S. Census Bureau Household Pulse Survey, Aug 2020 to Mar 2021 waves. Coefficients are derived from models in which the primary exposure is a triple interaction term between an indicator for whether the interview occurred after (versus before) the SNAP increase, a binary variable representing SNAP receipt, and a binary variable for whether the interviewee belonged to a given racial/ethnic or income group. All regressions adjust gender, age, marriage status, annual gross income, total household size, race, ethnicity, education, any work loss during COVID-19, fixed effects for survey week and state of residence, and robust standard errors. Binary outcome results represented as percentage points.

eTable 4. Effect of 15% SNAP benefit increase on health outcomes, using state-specific SNAP income eligibility criteria

	**Nutrition-related outcomes**			**Mental Health Outcomes**		**Secondary Outcomes**	
	**Household food insufficient**	**Children not eating b/c can't afford food**	**Free groceries/meals**	**Anxiety Symptoms Score (GAD-2)**	**Depressive Symptoms Score (PHQ-2)**	**Somewhat/very difficult paying expenses**	**Caught up rent/ mortgage**
**Coefficient**	-1.6**	0.4	-0.3	-0.07**	-0.02	-1.9*	-0.1
**[95% CI]**	[-2.9, -0.3]	[-1.8, 2.6]	[-1.5, 0.8]	[-0.1, -0.02]	[-0.08, 0.03]	[-3.1, -0.6]	[-1.2, 1.4]
**Observations**	96,438	34,235	96,495	96,363	96,314	96,530	73,506

Note: **p < 0.01, *p<0.05.
Abbreviations: GAD-2, Generalized Anxiety Disorder 2-item scale; PHQ-2, Patient Health Questionnaire 2-item scale, SNAP; Supplemental Nutrition Assistance Program
Data drawn from the U.S. Census Bureau Household Pulse Survey, Aug 2020 to Mar 2021 waves. Estimates represent the coefficient on the interaction term from difference-in-differences models adjusted for gender, age, marital status, income, household size, race/ethnicity, education, and work loss during COVID-19, as well as fixed effects for state and survey week, with robust standard errors. Binary outcome results reported as percentage points.

eTable 5. Effect of 15% SNAP benefit increase using alternative cut-offs for binary variables in sensitivity analyses

	**High/Very High Food Insufficiency**	**Very High Food Insufficiency**	**Anxiety Symptoms (GAD-2 Score ≥ 3)**	**Depressive Symptoms (PHQ-2 Score ≥ 3)**
Coefficient<br>[95% CI]	-0.3<br>[-2.0, 1.4]	-0.4<br>[-1.4, 0.6]	-1.6<br>[-3.5, 0.4]	-0.9<br>[-2.8, 1.0]

Note: **p < 0.01, *p<0.05.
Abbreviations: GAD-2, Generalized Anxiety Disorder 2-item scale; PHQ-2, Patient Health Questionnaire 2-item scale, SNAP; Supplemental Nutrition Assistance Program
Data drawn from the U.S. Census Bureau Household Pulse Survey, Aug 2020 to Mar 2021 waves. Estimates represent the coefficient on the interaction term from difference-in-differences models adjusted for gender, age, marital status, income, household size, race/ethnicity, education, and work loss during COVID-19, as well as fixed effects for state and survey week, with robust standard errors. Binary outcome results reported as percentage points.

eTable 6. Effect of 15% SNAP benefit increase intent-to-treat analysis

	Nutrition-related outcomes			Mental Health Outcomes		Secondary Outcomes	
	**Household food insufficient**	**Children not eating b/c can't afford food**	**Free groceries/meals**	**Anxiety Symptoms Score (GAD-2)**	**Depressive Symptoms Score (PHQ-2)**	**Somewhat/very difficult paying expenses**	**Caught up rent/ mortgage**
**Coefficient**	0.4	-0.1	-0.6	0.01	-0.02	-1.1*	-0.6
**[95% CI]**	[-0.4, 1.2]	[-1.7, 1.5]	[-1.2, 0.001]	[-0.02, 0.05]	[-0.05, 0.01]	[-1.8, -0.3]	[-1.3, 0.2]
**Observations**	230,304	56,373	230,383	230,067	229,980	230,515	171,357

Note: **p < 0.01, *p<0.05.
Abbreviations: GAD-2, Generalized Anxiety Disorder 2-item scale; PHQ-2, Patient Health Questionnaire 2-item scale, SNAP; Supplemental Nutrition Assistance Program
Data drawn from the U.S. Census Bureau Household Pulse Survey, Aug 2020 to Mar 2021 waves. Estimates represent the coefficient on the interaction term from difference-in-differences models adjusted for gender, age, marital status, income, household size, race/ethnicity, education, and work loss during COVID-19, as well as fixed effects for state and survey week, with robust standard errors (N=230,953).
Binary outcome results reported as percentage points.
